# Weak Acids as Endogenous Inhibitors of the Proton-Activated Chloride Channel

**DOI:** 10.3390/cells14141110

**Published:** 2025-07-19

**Authors:** Inês C. A. Pombeiro Stein, Maren Schulz, Daniel Rudolf, Christine Herzog, Frank Echtermeyer, Nils Kriedemann, Robert Zweigerdt, Andreas Leffler

**Affiliations:** 1 Department of Anesthesiology and Intensive Care Medicine, Hannover Medical School, Carl-Neuberg-Str. 1, 30625 Hannover, Germany; stein.ines@mh-hannover.de (I.C.A.P.S.); herzog.christine@mh-hannover.de (C.H.); echtermeyer.frank@mh-hannover.de (F.E.); 2 PRACTIS Clinician Scientist Program, Dean’s Office for Academic Career Development, Hannover Medical School, Carl-Neuberg-Str. 1, 30625 Hannover, Germany; 3 Leibniz Research Laboratories for Biotechnology and Artificial Organs (LEBAO), Department of Cardiothoracic, Transplantation and Vascular Surgery (HTTG), REBIRTH—Research Center for Translational Regenerative Medicine Hannover Medical School (MHH), Carl-Neuberg-Str. 1, 30625 Hannover, Germany; nils@kriedemann.net (N.K.);

**Keywords:** PAC, weak acids, PIP2, acidosis, chloride channel, cytotoxicity, binding site

## Abstract

The recently identified proton-activated chloride (PAC) channel is ubiquitously expressed, and it regulates several proton-sensitive physiological and pathophysiological processes. While the PAC channel is activated by strong acids due to the binding of protons to extracellular binding sites, here, we describe the way in which weak acids inhibit the PAC channel by a mechanism involving a distinct extracellular binding site. Whole-cell patch clamp was performed on wildtype HEK293T cells, PAC-knockout HEK293 cells expressing human (h)PAC mutant constructs, and on hiPSC-derived cardiomyocytes. Proton-induced cytotoxicity was examined in HEK293T cells. Acetic acid inhibited endogenous PAC channels in HEK 293T cells in a reversible, concentration-dependent, and pH-dependent manner. The inhibition of PAC channels was also induced by lactic acid, propionic acid, itaconic acid, and β-hydroxybutyrate. Weak acids also inhibited recombinant wildtype hPAC channels and PAC-like currents in hiPSC-derived cardiomyocytes. Replacement of the extracellular arginine 93 by an alanine (hPAC–Arg93Ala) strongly reduced the inhibition by some weak acids, including arachidonic acid. Although lactic acid inhibited PAC, it did not reduce the proton-induced cytotoxicity examined in wildtype HEK 293 cells. To conclude, weak acids inhibit PAC via an extracellular mechanism involving Arg93. These data warrant further investigations into the regulation of the PAC channel by endogenous weak acids.

## 1. Introduction

After the first functional description of its activity in cultured rat Sertoli cells in 2003 [1], the proton-activated chloride (PAC) channel was recently cloned and found to be expressed in several cell types [2,3,4]. The functional characteristics of the PAC channel include an outwardly rectifying single channel conductance and a voltage-dependent facilitation [5]. The PAC channel is also referred to as TMEM206 [2], the acid-sensitive outwardly rectifying anion channel (ASOR) [6], or the proton-activated outwardly rectifying anion channel (PAORAC) [5]. PAC is expressed on the plasma membrane, but PAC can traffic to the endosomes via YxxL-mediated endocytosis [7]. It has been shown that PAC mediates proton-induced cell swelling and cell death [2,3,4,8,9] in addition to macropinosome shrinkage [10]. In endosomes, PAC regulates endosomal pH values and transferrin-receptor-mediated endocytosis [7]. The activity of PAC channel is temperature-dependent, i.e., the channel’s activity increases at high temperatures [6,11]. It has been proposed that the PAC channel acts in combination with acid-sensing ion channels (ASICs) and proton-sensitive G-protein-coupled receptors (GPCRs) as a pH-sensing mechanism during brain ischemia [12]. PAC is expressed during osteoclast differentiation and regulates endplate porosity, and its expression has been suggested to increase spinal pain in a rodent lower back pain model [13]. Zhang and colleagues explored the prognostic significance of PAC in cancer entities, finding that PAC mRNAs had a negative prognostic value for patients with hepatocellular carcinoma (HCC) [14]. PAC overexpression was also detected in osteosarcoma, with high levels correlating with a more advanced clinical stage and pulmonary metastases [15]. These diverse physiological functions suggest that the PAC channel can be a key ion channel for a plethora of human diseases. Therefore, selective inhibitors of the PAC channel may have therapeutic effects. Several synthetic substances with inhibitory effects on PAC currents have been described, including 4,40-diisothiocyanatostilbene-2,20-disulfonate [DIDS] [4], diphenylamine-2-carboxylic acid [DPC] [1], 5-nitro-2-[3-phenylpropylamino]-benzoic acid [NPPB] [16], pregnenolone sulfate [2], flufenamic acid [5], niflumic acid [16], glibenclamide [17], simvastatin [18], and phloretin [9]. When it comes to endogenous PAC inhibitors, extracellular phosphatidylinositol [4,5]-bisphosphate [PIP2] was found to inhibit the PAC channel [19]. In contrast, intracellular PIP2 appears to be crucial for PAC channel activation [20]. It was also shown that arachidonic acid could potently inhibit PAC-like currents in HeLa cells [21]. Recently, the role of transfer RNA as a physiological inhibitor of the PAC channel was described [22].

Strong systemic or cellular acidosis can arise from an elevation of endogenous weak acids, including lactic acid, carbonic acid, or the ketone body β-hydroxybutyrate. Weak acids do not fully dissociate in solution, enabling them to permeate the cell membrane and induce intracellular acidosis [23]. While protons activate the capsaicin receptor TRPV1 via extracellular binding sites [24], weak acids like lactate inhibit TRPV1 [25]. In contrast, weak acids sensitize and activate TRPV2, TRPV3, and TRPA1 [23,26,27]. In this report, we describe the way in which some weak acids inhibit the PAC channel by binding to an extracellular binding site that is known to be crucial for inhibition by PIP2 [19]. Thus, like several other proton-sensitive ion channels, the PAC channel seems to differentiate between strong and weak acids.

## 2. Materials and Methods

### 2.1. Cell Culture

Human embryonic kidney 293 (HEK293) T cells were maintained under standard cell culture conditions (5% CO_2_ at 37 °C) in Dulbecco’s modified Eagle medium (DMEM, Bio&Sell, Feucht, Germany) supplemented with 10% fetal bovine serum (FBS, Bio&Sell, Feucht, Germany), 1% penicillin/streptomycin (Bio&Sell, Feucht, Germany), and glutamine (Bio&Sell, Feucht, Germany). TMEM206-knockout HEK293 cells and human PAC cDNA were a kind gift from Thomas J Jentsch (Berlin, Germany). HEK293T and TMEM206-knockout HEK293 cells were transfected with hPAC-plasmids using jetPEI^®^ (Polyplus, Illkirch, France). Mutant constructs were generated by site-directed mutagenesis using a Quickchange lightning site-directed mutagenesis kit (Agilent, Waldbronn, Germany); the ensuing sequencing confirmed the desired amino acid exchange and excluded unwanted mutations. Cells were detached using phosphate-buffered saline (PBS, Bio&Sell, Feucht, Germany) and trypsin/EDTA (Bio&Sell, Feucht, Germany) and were seeded for the patch clamp experiments.

### 2.2. hiPSC Cardiomyocyte Cultivation, Directed Cardiac Differentiation, and Maturation

An hiPSC cell, Phoenix [28], was differentiated in suspension for cell-only aggregates in stir–spinner flasks, as previously described in [29]. For the patch clamp experiments, hiPSC cardiomyocytes were dissociated for 3 min using a STEMdiff Cardiomyocyte Dissociation Kit (StemCell Technologies, Cologne, Germany) after 14 to 18 days of differentiation. The dissociated cells were plated on fibronectin + 0.1% gelatine-coated round-glass coverslips in medium, containing 80% IMDM + Glutamax (GIBCO-Invitrogen, Darmstadt, Germany), 20% fetal calf serum, 1 mM L-glutamine, 0.1 mM ß-mercaptoethanol, and 1% nonessential amino acids (all GIBCO-Invitrogen). For the first 24 h, the medium was supplemented with 10 µM Y-27632 (ROCK inhibitor). Afterwards, the medium was replaced with RPMI-1640+B-27 supplement (ThermoFisher Scientific, Waltham, MA, USA), which was replenished every 2–3 days before the patch clamp experiments were performed at ~21 to 25 days post cell seeding [30,31].

### 2.3. Patch Clamp Electrophysiology

Whole-cell voltage clamp recordings were obtained by an EPC9 and EPC10 USB HEKA amplifier and HEKA Patchmaster software version v2x90.5 (HEKA Elektronik, Reutlingen, Germany). Pipettes were produced from borosilicate glass tubes (GB150TF-8P; Science Products, Hofheim, Germany) through a puller (Model PP-830, Narishige, Japan) to deliver a resistance of 3–5 MΩ when filled with intracellular solution. All experiments were conducted at room temperature. Only isolated, individual cells without connections to neighboring cells were utilized. A gravity-driven multi-barrel perfusion system allowed the application of solutions. During measurements, current traces recorded at +60 mV or +20 mV were low-pass-filtered using an analogue 1 kHz eight-pole Bessel filter and digitally filtered at 300 Hz, where the sampling rate was 10 kHz. Liquid junction potential was not corrected for. Current voltage curves were measured during 500 ms long voltage ramps from −100 to +100 mV, with a holding potential of −60 mV. For voltage-dependent activation, cells were held at −60 mV; 200 ms long voltage step pulses were applied from −60 mV to +120 mV in 20 mV increments.

The standard intracellular pipette solution contained (in mM): 140 CsCl, 2 MgCl_2_, 10 MES, and 5 EGTA. pH 7.2 or 5.0 was adjusted using CsOH. The standard external solution contained (in mM): 140 NaCl, 5 KCl, 2 MgCl_2_, 10 glucose, 10 MES, 5 EGTA. The pH of the solution was adjusted with NaOH to the desired values. We decided on MES as a buffering system in order to avoid the utilization of citrate; this is because citrate is a weak acid and could thus distort the results. Because the buffering capacity of MES is not ideal for the range of different pH levels used in this project, the pH value of all solutions (including internal solution) was controlled before the beginning of each experimental day. Pregnenolone sulfate, lactic acid, propionic acid, itaconic acid, flufenamic acid and niflumic acid were purchased from Sigma Aldrich/Merck (Darmstadt, Germany). Acetic acid was purchased from Carl Roth (Karlsruhe, Germany). All substances were dissolved according to manufacturers’ guidelines and diluted to provide appropriate concentrations. After the addition of the weak acids to the solutions, special care was taken to titrate the pH to the desired values in order to ensure that the correct experimental conditions were attained.

For data acquisition and offline analyses, PatchMaster/FitMaster v2x92 (HEKA Elektronik, Reutlingen, Germany) and Origin 8.5.1. software (OriginLab, Northampton, MA, USA) were used. All data are presented as mean ± SEM. Statistical analysis was performed with GraphPad Prism version 10.4.1 for Windows, GraphPad Software (Boston, MA, USA). Normally distributed data were analyzed by parametric testing (paired or unpaired students *t*-tests, one-way ANOVA followed by Tukey post hoc test); non-normally distributed data were analyzed by non-parametric testing (Mann–Whitney U test, Kruskal–Wallis ANOVA, followed by Dunn-Bonferroni post hoc test). Measurements were performed on at least two experimental days. In all figure legends, * denotes *p* < 0.05, ** denotes *p* < 0.01, and *** denotes *p* < 0.001.

### 2.4. Cell Death Experiments

Cell death experiments were conducted as previously described in [26]. Briefly, HEK293 cells were plated on rat tail collagen-coated slips (Corning, Kaiserslautern, Germany) and incubated under normal conditions for 3 days in order to ensure replication and adhesion. After 3 days, cells were treated with neutral, acidic, or lactate-containing acidic patch clamp buffers and incubated for 2 h at 37 °C. After acidic incubation, cells were either directly stained or maintained with DMEM at standard conditions, as described above, for a further 4 h. The cells were stained with 10 µg/mL propidium iodide (PI, Sigma-Aldrich/Merck, Darmstadt, Germany) for 20 min at 37 °C and afterwards fixated with 4% paraformaldehyde (PFA, ThermoFischer, Darmstadt, Germany) in PBS for 20 min; then, they were counterstained with 1 µg/mL 4′,6-Diamidin-2-phenylindol (DAPI, AppliChem, Darmstadt, Germany) in PBS with 1% bovine serum albumin (BSA, GE Healthcare, Pasching, Austria) for 20 min at room temperature. Images were acquired on an inverted microscope IX81 (Olympus K. K., Tokyo, Japan) with an Orca Flash 4.0 LT camera (Hamamatsu Photonics K.K., Shizuoka, Japan) and CellSense Dimension 3.2 software (Olympus K. K., Tokyo, Japan). Picture analyses (percentage of PI positive cells over total number of cells) were performed with ImageJ 1.54g (National Institutes of Health, Bethesda, MD, USA) and MS Excel (Microsoft Corporation, Washington, DC, USA).

## 3. Results

### 3.1. Effects of Acetic Acid on Endogenous PAC Channels in HEK 293T Cells

**Figure 1:**
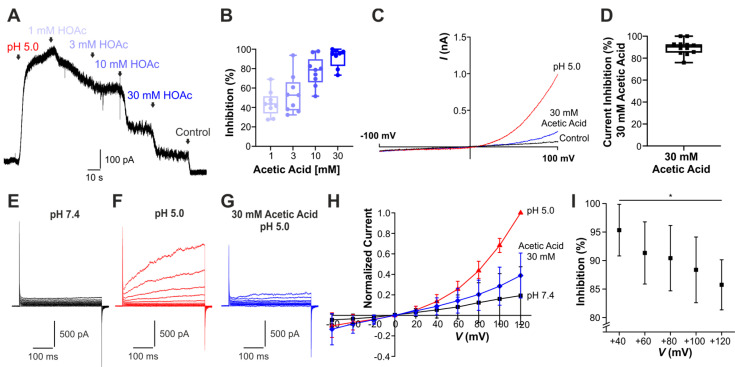
Acetic acid inhibits endogenous PAC-mediated currents in HEK293T cells. (**A**) Representative PAC current from HEK293T cells evoked by pH 5.0 and concentration-dependent inhibition by acetic acid (HOAc) at pH 5.0 (n = 9). (**B**) Box diagrams with dot plots displaying the induced current reduction by increasing concentrations of HOAc at pH 5.0 (n = 9 for each concentration). (**C**) Membrane current in a HEK293T cell elicited by pH 5.0 during 500 ms long ramps from −100 mV through 100 mV with control solution (pH 7.4), pH 5.0, or the combination of pH 5.0 and 30 mM HOAc (n = 11). (**D**) Box diagram with dot plots demonstrating that PAC currents elicited by voltage ramps are inhibited by 30 mM HOAc (n = 11). (**E**–**G**) Voltage-dependent membrane currents in HEK293T cells evoked by 200 ms long, 20 mV voltage step pulses from −60 mV through 120 mV at pH 7.4 (**E**), pH 5.0 (**F**), or after application of 30 mM HOAc at pH 5.0 (**G**) (n = 10). (**H**) Normalized currents evoked by voltage step pulses and application of either control solution (black), pH 5.0 (red), or the combination of 30 mM acetic acid and pH 5.0 (blue) (n = 10). (**I**) Current inhibition caused by co-application of pH 5.0 and 30 mM acetic acid is voltage-dependent, displaying smaller effects at more positive potentials, * *p* < 0.05 (one-way ANOVA, Tukey’s post hoc test).

Proton-induced (pH 5.0) outward currents were examined in cells held at +60 mV. As is demonstrated in Figure 1A,B, increasing concentrations of acetic acid titrated to pH 5.0 (HOAc, 1, 3, 10, and 30 mM, n = 9 for each concentration) induced a concentration-dependent inhibition of PAC-mediated currents. When pH 5.0-induced currents were monitored during 500 ms long ramps from −100 to 100 mV, we observed a strong inhibition by HOAc as well (Figure 1C,D, n = 11). In order to test the voltage dependency of this inhibition, 200 ms long voltage pulses were applied from −60 mV through 120 mV in steps of 20 mV (Figure 1E–G, n = 10). As shown in Figure 1H,I, the current inhibition exerted by acetic acid is indeed voltage-dependent, with smaller effects being observed at more depolarized potentials.

### 3.2. Effects of More Physiologically Relevant Weak Acids

**Figure 2:**
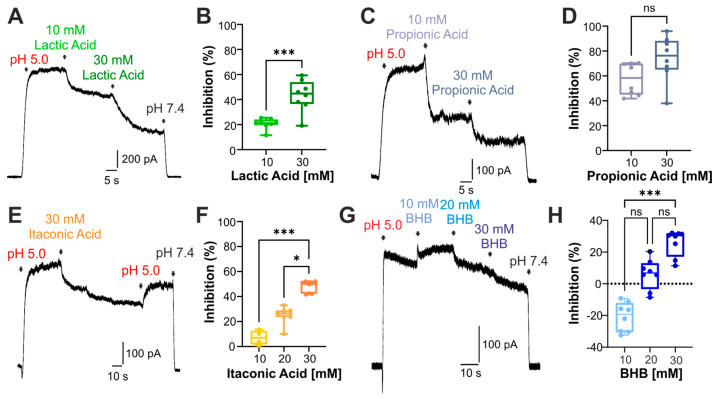
Endogenous weak acids inhibit PAC-mediated currents in HEK293T cells. (**A**) HEK293T cells held at +60 mV generate a PAC-induced current which is inhibited by increasing concentrations of lactic acid at pH 5.0 (n = 8). (**B**) Box diagram with dots displaying the concentration-dependent current inhibition by lactic acid (unpaired *t*-test; *** denotes *p* < 0.001). (**C**) Representative PAC current and its inhibition by increasing concentrations of propionic acid (n = 8). (**D**) Box diagram with dots demonstrating the current inhibition by propionic acid (Mann–Whitney U test; ns denotes not significant). (**E**) PAC-mediated current inhibited by 30 mM itaconic acid at pH 5.0. Termination of itaconic acid and application of solely pH 5.0 induced a partial recovery of the PAC-invoked current (n = 8). (**F**) Box diagram with dots illustrating the concentration-dependent current inhibition by a combination of pH 5.0 and itaconic acid (Kruskal–Wallis ANOVA with Dunn–Bonferroni post hoc test, * denotes *p* < 0.05, and *** denotes *p* < 0.001, n = 4–8). (**G**) PAC-mediated current inhibition by increasing concentrations of *β*-hydroxybutyrate (BHB) (n = 8). (**H**) Box diagram with dots illustrating the concentration-dependent current inhibition by *β*-hydroxybutyrate (Kruskal–Wallis ANOVA with Dunn–Bonferroni post hoc test; *** denotes *p* < 0.001).

Similar to acetic acid, we observed that lactic acid (Figure 2A,B, n = 8), propionic acid (Figure 2C,D, n = 8), and itaconic acid (Figure 2E,F, n = 8) also inhibited endogenous PAC-like currents in a concentration-dependent manner at pH 5.0. While 10 mM *β*-hydroxybutyrate (BHB) slightly increased pH 5.0-evoked currents, we observed a modest current inhibition by 20 and 30 mM BHB (Figure 2G,H, n = 8).

**Figure 3:**
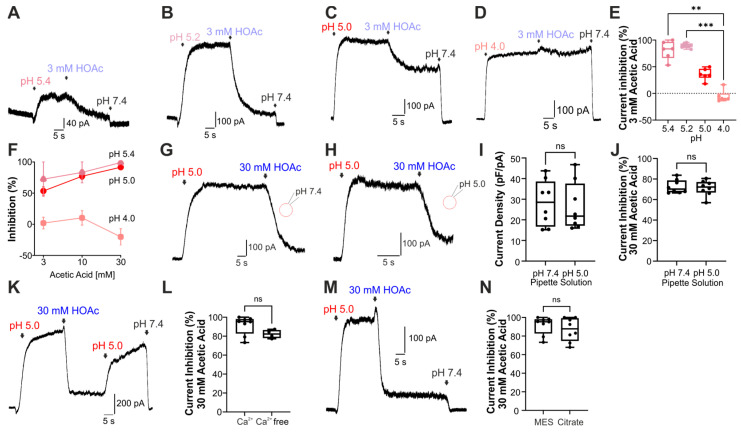
Properties of acetic acid sensitivity of endogenous PAC channels. (**A**–**D**) Representative PAC currents from a HEK293T cells held at +60 mV. Currents were evoked by pH 5.4 ((**A**), n = 7), pH 5.2 ((**B**), n = 7), pH 5.0 ((**C**), n = 5), or pH 4.0 ((**D**), n = 6). (**E**) Box diagram with dot plots representing PAC current inhibition by 3 mM HOAc at different pH values (Kruskal–Wallis ANOVA with Dunn–Bonferroni post hoc test; ** denotes *p* < 0.01; *** denotes *p* < 0.001). (**F**) Concentration- and pH-dependency of the inhibition of PAC by HOAc (n = 7–9 cells for each concentration and pH value). (**G**,**H**) Current traces demonstrating inhibition of PAC-mediated currents by 30 mM HOAc at pH 5.0 using pipette solutions with pH 7.4 ((**G**), n = 8) or pH 5.0 ((**H**), n = 8). (**I**,**J**) Acidification of the cytoplasm with pH 5.0 neither had an influence on the current density of PAC-mediated currents (**I**), nor on the inhibitory effect of 30 mM HOAc (**J**) (unpaired *t*-test, ns denotes not significant). (**K**) PAC-mediated current induced by pH 5.0 with an extracellular solution containing 2 mM calcium (n = 4). (**L**) Box diagram with dots representing the current inhibition by 30 mM HOAc in calcium-containing and calcium-free extracellular solutions (n = 4 and 9; Mann–Whitney U test; ns denotes not significant). (**M**) Current from a HEK293T cell recorded in citrate-buffered extracellular solution (n = 8). (**N**) Box diagram with dots representing the current inhibition by 30 mM HOAc in MES or citrate-buffered extracellular solution (n = 8 cells for each condition; Mann–Whitney U test; ns denotes not significant).

In order to explore the mechanism responsible for this inhibition of the PAC channel by weak acids, we next performed experiments that allowed us to differentiate between extracellular and intracellular mechanisms. To explore the pH-dependency of the HOAc-induced inhibition of the PAC channel, current inhibition with a defined HOAc concentration (3 mM) at different pH values (n = 6–8 for each pH level) was determined. Cells were held at +60 mV and solutions with pH 4.0, 5.0, 5.2, or 5.4 were applied (Figure 3A–D). As demonstrated by Figure 3E,F, the ability of HOAc to inhibit PAC-like currents diminished with lower pH values. At pH 4.0, the application of 3 mM HOAc could even augment the PAC-mediated current (Figure 3D–F). We next sought to determine whether the weak-acid-induced inhibition is simply due to intracellular acidification. The internal patch pipette solution was titrated to pH 5.0 and measurements were started 3 min after establishing whole cell configuration, enabling the full adjustment of the cytosolic pH value (Figure 3G,H). As shown by Figure 3I, a more acidic pipette solution did not influence the elicited current densities of pH-induced currents (n = 8). Furthermore, inhibition by 30 mM HOAc was not altered with an acidified pipette solution (Figure 3J, n = 8). To our surprise, these data suggest that the observed inhibition of PAC-mediated currents occurs through an extracellular rather than an intracellular mechanism. Our laboratory recently demonstrated that extracellular calcium effectively inhibits HOAc-induced activation of TRPV2 [26], and lactic acid potentiates proton-induced activation of acid-sensitive ion channels (ASICs) by chelating calcium ions that compete with protons in extracellular bindings sites [32]. As we performed our experiments in nominal calcium-free solutions, we sought to determine whether the presence of 2 mM calcium in the extracellular solution (i.e., more physiological conditions) has an impact on the observed weak-acid-induced inhibition of PAC. However, calcium had no effect on HOAc-induced current inhibition (Figure 3K,L, n = 4). To rule out that the use of MES as a buffering system influenced the inhibition of PAC-induced currents by HOAc, we repeated the experiments with 30 mM HOAc in citrate-buffered solutions. As is demonstrated in Figure 3M and N (n = 8 for each condition), we could not observe relevant differences.

**Figure 4:**
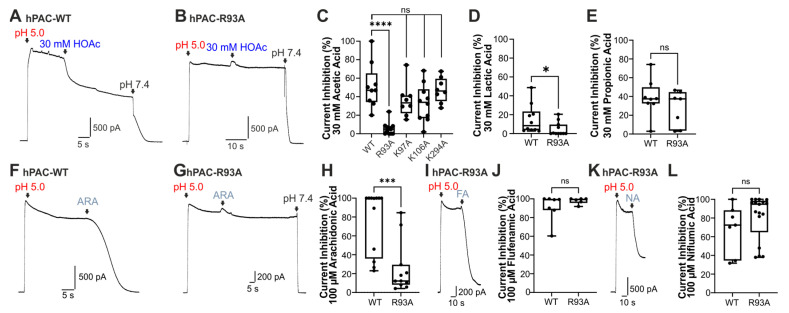
Reduced inhibition by weak acids on hPAC–Arg93Ala. (**A**) Typical recordings on PAC-knockout HEK293 cells transfected with hPAC-WT, showing a representative PAC-mediated current in a cell held at +20 mV upon stimulation with pH 5.0 and inhibition by 30 mM HOAc (n = 9). (**B**) Current mediated by hPAC–Arg93Ala expressed in PAC-knockout HEK293 cells. Note that 30 mM HOAc failed to inhibit this mutant (n = 11). (**C**) Box diagram with dots displaying the current inhibition induced by 30 mM HOAc in (**A**,**B**) and further mutant hPAC constructs (Kruskal–Wallis ANOVA with Dunn–Bonferroni post hoc test; ns denotes not significant; **** denotes, *p* < 0.0001). (**D**) Box diagram with dots demonstrating the significantly reduced current inhibition by 30 mM lactic acid on hPAC–Arg93Ala (Mann–Whitney U test; * denotes *p* < 0.05; n = 12). (**E**) Box diagram with dots showing that inhibition of hPAC–Arg93Ala by 30 mM propionic acid was not significantly reduced (Mann–Whitney U test; n = 8). (**F**,**G**). Typical currents generated by hPAC-WT ((**F**), n = 12) and hPAC–Arg93Ala ((**G**), n = 11). While hPAC-WT was almost completely inhibited by 30 µM arachidonic acid (ARA) at pH 5.0, inhibition of hPAC–Arg93Ala was strongly reduced. (**H**) Box diagram with dots demonstrating the significantly reduced current inhibition of hPAC–Arg93Ala by 30 µM arachidonic acid (Mann–Whitney U test; *** denotes *p* < 0.001). (**I**) Typical current generated by hPAC–Arg93Ala (n = 6) at +20 mV, displaying a strong inhibition by 100 µM flufenamic acid (FA). (**J**) Box diagram with dots demonstrating that flufenamic acid inhibits hPAC-WT and hPAC–Arg93Ala to a similar degree (Mann–Whitney U test; ns denotes not significant). (**K**) Typical current generated by hPAC–Arg93Ala (n = 17) at a holding potential of +20 mV, displaying a strong inhibition by 100 µM niflumic acid (NA). (**L**) Box diagram with dots illustrating that the current inhibition induced by 100 µM niflumic acid did not differ between hPAC-WT and hPAC–Arg93Ala (Mann–Whitney U test; ns denotes not significant).

We next sought to determine whether weak acids interact with the positively charged extracellular residues that were demonstrated to be crucial for the inhibition of the PAC channel by PIP2 [19]. We replaced the responsible residues, Arg93, Lys97, Lys106, and Lys294, with alanine (Ala) through directed mutagenesis and transfected TMEM206-knockout HEK293 cells with either wildtype or mutated hPAC constructs. Because of the very large current produced by transfected cells and consequent cell death, which rendered measurements impossible, transfected cells were held at +20 mV. As is shown in Figure 4A, the current elicited in cells with wildtype hPAC was effectively inhibited by 30 mM HOAc (n = 9). This inhibition was similar to that observed in wildtype HEK293T cells. In contrast, currents generated by cells transfected with hPAC–Arg93Ala showed largely no inhibition by 30 mM HOAc (Figure 4B,C, n = 11). The mutants hPAC–Lys97Ala, -Lys106Ala, and -Lys294Ala showed no statistically significant different inhibition by 30 mM HOAc as compared to hPAC-WT (Figure 4C, n = 8–10 for each phenotype). We also explored the effects of lactic acid and propionic acid on hPAC–Arg93Ala. While lactic acid almost completely failed to inhibit hPAC–Arg93Ala (Figure 4D, n = 12), inhibition by propionic acid was similar in hPAC-WT and hPAC–Arg93Ala (Figure 4E, n = 6 and 17). Several substances that were previously demonstrated to potently inhibit PAC-like currents are weak acids, including arachidonic acid, flufenamic acid, and niflumic acid, which were reported to inhibit PAC-like currents at low micromolar concentrations [21]. To this end, we sought to determine whether the extracellular residue Arg93 is relevant for inhibition of these substances as well. We examined the effect of saturating concentrations of arachidonic acid (30 µM, Figure 4E–G, n = 12 and 11), flufenamic acid (100 µM, Figure 4H,I, n = 7 and 6), and niflumic acid (100 µM, Figure 4J,K, n = 7 and 15). While inhibition by flufenamic and niflumic acid was not reduced on hPAC–Arg93Ala, arachidonic acid almost completely failed to inhibit PAC-Arg93Ala.

### 3.3. Generation of PAC-like Currents by hiPSC-Derived Cardiomyocytes and Cell Death Experiments

**Figure 5:**
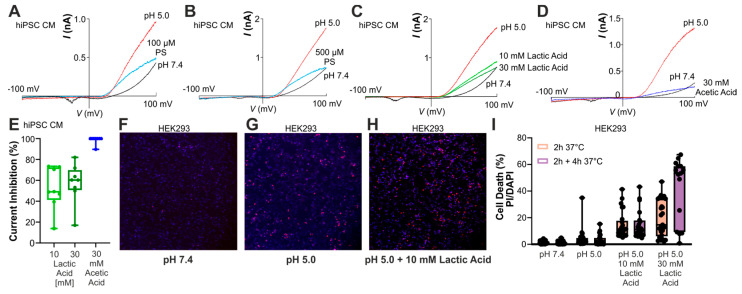
hiPSC-derived cardiomyocytes generate PAC-like currents, which are inhibited by weak acids. (**A**,**B**) Membrane currents in hiPSC-derived cardiomyocytes challenged with pH 5.0 or the combination of pH 5.0 and (**A**) 100 µM pregnenolone sulfate (PS, n = 6), or (**B**) 500 µM pregnenolone sulfate (n = 7 cells). (**C**,**D**) Membrane currents in hiPSC-derived cardiomyocytes evoked by pH 5.0 or the combination of pH 5.0 and 10 or 30 mM lactic acid ((**C**), n = 8), or 30 mM HOAc ((**D**), n = 8). (**E**) Box diagram with dots representing the PAC current inhibition by different concentrations of lactic (n = 8) and acetic acid (n = 8). (**F**–**H**) Representative figures of HEK293 cells following treatment with pH 7.4 (**F**), pH 5.0 (**G**), or pH 5.0 and 10 mM lactic acid (**H**) for 2 h and subsequent incubation with standard medium for 4 h at 37 °C. Cells were double-stained with DAPI (blue) and PI (red). (**I**) Box diagrams with dots displaying the percentage of dead cells (PI-positive) over absolute (DAPI-positive) cell count for two experimental conditions (2 h 37 °C or 2 h 37 °C and subsequent 4 h standard cell culture incubation; each condition n = 5 with independent experiments).

We next aimed to determine whether the inhibition of the PAC channel by weak acids can also be observed in cells other than HEK293 cells, and we investigated human-induced pluripotent stem cell-derived (hiPSC) cardiomyocytes. At pH 7.4, these cells produced small outward currents at positive membrane potentials, obviously generated by voltage-gated channels of unknown identity. Upon application of pH 5.0, hiPSC-derived cardiomyocytes produced large PAC-like currents (Figure 5A, n = 6). The PAC-inhibitor pregnenolone sulfate effectively inhibited the pH 5.0-induced increase in outward currents (Figure 5A,B, n = 6–7 cells), suggesting that they are indeed mediated by PAC [2]. Accordingly, both lactic acid (Figure 5C,E, n = 8) and HOAc (Figure 5D,E, n = 8) reduced the outward currents induced by pH 5.0. Although this reduction may also include an inhibition of the voltage-gated channels observed at pH 7.4, the data suggest that endogenous PAC channels in hiPSC-derived cardiomyocytes are blocked by weak acids.

Because of the previously described role of PAC in acid-induced cell death and ischemic injury [3], we finally sought to determine whether the inhibition of PAC by lactic acid—known to accumulate during ischemia [33]—results in a protective effect. The effect of lactic acid on the cytotoxicity of an acidic stimulus (pH 5.0) was examined on wildtype HEK293T cells. Cell death following incubation at pH 5.0 for 2 h was marginal (Figure 5F–I). When 10 and 30 mM lactic acid were added to the media, we observed a concentration-dependent augmentation (instead of a reduction) of this acidotoxicity (Figure 5F–I). To investigate the influence of an eventual ischemia/reperfusion injury, we simulated reperfusion by maintaining the cells for another 4 h under standard cell culture conditions. This model for reperfusion injury indeed induced an increase in cytotoxicity, but again lactic acid even aggravated this effect (Figure 5I).

## 4. Discussion

The molecular identity of the PAC channel, the ion channel that mediates proton-activated chloride currents, was only recently revealed [2,3]. The almost ubiquitous expression of the PAC channel suggests that it may have fundamental physiological and pathophysiological roles, raising the possibility that the PAC channel could serve as an important pharmacological target for the treatment of human diseases. The basic functional and structural properties of the PAC channel have already been characterized in detail [2,3,16,34], but little is known about endogenous molecules that can functionally modify the PAC channel. In our study, we demonstrate that weak acids inhibit the PAC channel in a reversible and concentration-dependent manner. This effect is due to the interaction of weak acids with an extracellular site of the PAC channel, and we found that a previously identified binding site [Arg93] for PIP2 also appears to be crucial for inhibition by some weak acids [19]. Although the activation of the PAC channel by strong acidosis is considered to be an important mechanism in acidotoxicity [2,3,11], our in vitro data suggest that the inhibition of the PAC channel by lactic acid does not reduce proton-induced cell death.

Mihaljević and colleagues proposed that the negative charge in combination with stable insertion into the membrane of PIP2 are likely to be favorable pharmacological characteristics of PAC inhibitors [19]. Depending on the pH value of the solution and pK_a_ value, weak acids partially dissociate into a conjugate of a negatively charged base and a proton. Furthermore, weak acids were described to induce an increase in lipid solubility, to change membrane elasticity and stiffness, and to dissipate the proton motive force across the membrane [35,36,37]. Similar to PIP2, weak acids may therefore fulfill both of the suggested attributes required for the inhibition of the PAC channel. In this study, we mainly used acetic acid to characterize the weak-acid-induced block of the PAC channel. With a pK_a_ of 4.76, 64% of acetic acid is protonated and is, therefore, membrane-impermeable at pH 5.0. This fraction changes to 15% at pH 4.0, and to 73% at pH 5.2. We found that the inhibition of the PAC channel was more effective at pH 5.2 than at pH 5.0, but we did not observe any robust inhibition at pH 4.0. Furthermore, intracellular acidification neither inhibited the activation of the PAC channel by extracellular protons nor had an effect on the inhibition by acetic acid. These data indicate that acetic acid inhibits the PAC channel by employing an extracellular mechanism. We are only aware of one known extracellular mechanism for the inhibition of the PAC channel, i.e., the recently described inhibition of the PAC channel by extracellular PIP2 [19]. By analyzing a Cryo-EM structure of PIP2-bound PAC, the authors identified and functionally validated binding of PIP2 on a number of positively charged extracellular residues located between transmembrane domains 1 and 2 [19]. Negatively charged phosphate groups of the inositol head of PIP2 were found to form a salt bridge with the residue Arg93, and the lipophilic acyl with hydrophobic residues. We found that the inhibition of the PAC channel by acetic, lactic, and arachidonic acids was strongly reduced in the hPAC–Arg93Ala mutant. Replacement of the closely adjacent positively charged residues Lys97, Lys106, and Lys294 by alanine did not result in a statistically significant reduction in inhibition; but, at least for hPAC–Lys97Ala and PAC–Lys106Ala, our data suggest a small reduction in the inhibition achieved by acetic acid. When compared to the large molecule PIP2 with a molecular weight of >100 Da, the weak acids studied in this project are small molecules with molecular weights < 100 Da, except for arachidonic acid, with a molecular weight of 304 Da. Therefore, the mechanisms for binding on PAC cannot be expected to fully overlap. Nevertheless, our functional data strongly suggest that Arg93 is a key extracellular site on human PAC for inhibition by at least some weak acids. In contrast to what we observed for inhibition by acetic acid, the ability of PIP2 to inhibit the PAC channel was described to increase at lower pH values [19]. The authors correlated this effect to the degree of acute desensitization observed at low pH values for PAC in that study [19]. We did not observe this prominent desensitization of PAC-mediated currents even at pH 4.0. Therefore, we cannot say whether weak acids have an impact on the desensitization of PAC as well.

In order to broaden the scope of our study towards the question of the physiological relevance of the weak-acid-induced inhibition of the PAC channel, we also performed experiments on hiPSC-derived cardiomyocytes. We could observe that PAC-like proton-evoked currents in these cells could be inhibited by the known PAC-blocker pregnenolone sulfate, as well as weak acids. Since ischemia with subsequent acidosis and formation of lactic acid under anaerobic conditions plays an important role in cardiac pathologies, our data place PAC as a possible molecular determinant for the effects of both protons and lactic acid in this process. Furthermore, previous reports found that endogenous PAC channels in HEK293 cells are relevant for proton-induced cell death [2,3,11]. We only observed marginal acidotoxicity when HEK293 cells were exposed to pH 5.0, making it challenging to detect the expected partial reduction by weak acids. Surprisingly, we even observed a prominent increase in cell death when lactic acid was added to the medium. It is possible that this increase in cytotoxicity is mediated by endogenous acid sensing ion channels (ASICs) that are known to mediate acidotoxicity, and to be potentiated by lactic acid [32,38,39]. It was beyond the scope of this initial report to establish whether this hypothesis stands true; it was also beyond the present scope to conduct further experiments examining a possibly protective effect of other weak acids in models of PAC-mediated acidotoxicity. To this end, our data, demonstrating that high concentrations of β-hydroxybutyrate also inhibit the PAC channel, might warrant further investigations. β-hydroxybutyrate and other ketone bodies have been demonstrated to be neuroprotective and to mitigate cellular damage following ischemia reperfusion in several organs [40,41]. Unlike lactic acid, β-hydroxybutyrate even seems to inhibit ASICs [42]. Therefore, it seems plausible that a combined suppression of ASICs and the PAC channel during acidosis might be relevant for cellular protection accomplished by β-hydroxybutyrate.

Another finding that warrants further investigations is the property of itaconic acid as an inhibitor of the PAC channel. High concentrations of itaconic acid are released from the mitochondria of activated macrophages, and it seems to exert several anti-inflammatory effects, including an inhibition of the late inflammasome and an increase in interferon type 1 secretion [43,44]. Interestingly, a recent study on PAC-knockout mice found that macrophages lacking PAC exhibit an augmented phenotype with a strong increase in interferon response as well as in inflammasome activation [45]. Further studies are required to establish whether or not an interaction between the PAC channel and itaconate is relevant.

## 5. Conclusions

Taken together, we demonstrate an inhibition of the PAC channel by weak acids and identify a key extracellular residue of PAC for this channel modulation. Our data lay a foundation for further studies on the relevance of this property for the protective effects of lactic acid and other weak acids against acidotoxicity. Furthermore, investigations on the likely relevance of Arg93 as a common binding site for charged modulators of the PAC channel seem warranted.

## Data Availability

All data are presented in the manuscript. Original data can be obtained from corresponding authors upon reasonable request.

## Abbreviations

The following abbreviations are used in this manuscript:

ARA	arachidonic acid
ASIC	acid-sensing ion channels
ASOR	acid-sensitive outwardly rectifying anion channel
BSA	bovine serum albumin
BHB	β-hydroxybutyrate
DAPI	4′,6-Diamidin-2-phenylindol
DPC	diphenylamine-2-carboxylic acid
DIDS	4,40-diisothiocyanatostilbene-2,20-disulfonate
DMEM	Dulbecco’s modified Eagle’s medium
EDTA	ethylene diamine tetra acetic acid
FBS	fetal bovine serum
GPCR	G-protein-coupled receptors
HCC	hepatocellular carcinoma
HEK	human embryonic kidney
hiPSC	human-induced pluripotent stem cell
HOAc	acetic acid
NPPB	5-nitro-2-(3-phenylpropylamino)-benzoic acid
PAC	proton-activated chloride channel
PAORAC	proton-activated outwardly rectifying anion channel
PBS	phosphate-buffered saline
PFA	Paraformaldehyde
PI	propidium iodide
PIP2	phosphatidylinositol (4,5)-bisphosphate
PS	pregnenolone sulfate
TMEM	transmembrane proteins
WT	wildtype
